# The Function and Value of the Dental Society

**Published:** 1903-04-15

**Authors:** N. S. Hoff


### THE FUNCTION AND VALUE OF THE DENTAL SOCIETY.

BY N. S. HOFF, D.D.S.

Read before the Detroit Dental Society, December 11, 1902.

It would be interesting and helpful, had we the time to review the history of the inception and development of the dental social organization, which we see to-day everywhere exercising so helpful an influence in elevating professional standards and is doing so much to harmonize the rules of accepted practice. Before the era of social organizations dawned, our profession was little better than a trade: secrecy and even superstition abounded to a degree now incomprehensible. Fortunately a few wise and far seeing choice spirits as early as 1840, believed that the profession was destined to a nobler fate than to be buried in the laboratories of a few selfish and short sighted practitioners who were not far removed from the charlatans of to-day, and who persisted in acting upon the theory that to give up their method of practice was to surrender forever all chances for maintaining their supremacy in practice,

Although several local organizations existed in the Eastern states, no national or even local organization of any considerable notice had been affected until a convention of dentists met in New York City the 18th of August, 1840, and organized what was called the American Society of Dental Surgeons. This society for four years kept the place of a national organization, but owing to the great distances and difficulties of travel at that time it was deemed expedient by the Western dentists to organize a society which could be more readily reached by the Mississippi Valley dentist and in 1844 the Mississippi Valley Dental Society was organized. From this time and from the influence exerted by the members of these organizations, the dental society became a popular and increasing force in the cultivation of that skill which has made the American dentist a professional man known and esteemed the world over. We have no data as to the number of dental societies in our country at the present time, but it is safe to say that the progressive element of our profession will be found in some of the hundreds of local, State and national organizations of the present times.

The question that is often asked is why are not all dentists members of and active participants in some one dental society at least. We prefer at present not to attempt to reply to this question as we are not sufficientily in sympathy with this class of dentists to make a satisfactory reply. We have preferred to approach the subject from a standpoint more in harmony with our own experience and ideals, and will endeavor to portray some of the attractive features which have drawn us into society work, and which make it worthy of our highest esteem.

Therefore, it will be well for us to examine the dental society from the general standpoint of what it is doing as an organism in the development of the profession. And the first and perhaps the most striking feature which commands notice is its literary output. If we turn to our periodic literature for evidence we shall very soon discover that by far the largest amount of original matter comes to the journals not as contributed articles, but as papers read and discussed in some dental society. In verification of this assertion we find on examination of a recent periodical of the best class that it contains ten pages, including editorial, of original contributed matter to eighty-four pages of original matter obtained through dental societies.

The reason for this is evident. It shows also instead of the producers of new ideas and methods endeavoring to conceal them as in old times, they are now glad to present them to their confreres for criticism or amplification, as may be the case, in the open discussion following their reading. It may be true as is often said that many of these papers would never had been written had it not been for the importunities of zealous program committeemen. This is very likely, but it is a blessing that works no harm even if the papers happen to be void of originality, or lacking in practical significance. It generally happens that they serve the purpose of a text at least, which draws out latent ideas of practical significance and thus justify their conception and the necessity for the organization through which they obtain this public consideration. So that for this reason alone we shall claim the dental society has earned a right to our cordial endorsement as a useful and valuable institution for the dissemination of literature and for the intellectual development of the profession.

If we again consider its function as a medium for developing the technical aspect of our profession we shall readily concede to it a high place. We recently attended the meeting of one of our prominent State societies and three of the eight papers read were on strictly technical subjects, and three others were on correlated subjects. There were twenty-four clinics and between twenty-five and thirty exhibits of technical appliances. This illustration may be duplicated in hundreds of meetings, except that in the majority the technical subjects discussed in the papers will predominate rather than be in the majority as happened in this case. The program of a meeting of prominent society soon to meet offers but four papers, and a clinic of more than one hundred participants. (This meeting has been held since this paper was written and was attended by twenty-eight hundred dentists, the largest attendance ever recorded—ED.) It may be said that the present tendency in dental societies to allow the technical features to so largely predominate is a sympton of retrogression, which will run its course or bankrupt the society proceedings from a literary standpoint. Whilst this danger may be possible, it still remains true that these features have always been important adjuncts of society meetings and they do not seem to have detracted from the literary or scientific attainments of regular society attendants. The fact is that in these meetings all technical exhibits undergo the sharp criticism of the best and most experienced thinkers and technical workers. And this quality of investigation leads not only to the modification of faulty methods and appliances from the factor's standpoint, but to a closer scrutiny also of technical details by men of limited skill in devising or choosing methods and facilities suited to their own peculiar environments. It is a kind of reflex movement by which men are stimulated to not only surround themselves with appliances of other's devising, but it also creates a taste for inventing new methods and appliances for themselves. It keeps one's wits on the alert to seize advantages made possible by others if not of his own initiative, and this prevents stagnation, or what is worse, following in the humdrum of a narrow groove, which dwarfs the intellect, makes the heart sick, and shrivels the soul by the continued repetition of an endless routine. It may be truthfully said that the advances in technical departments find their inspiration very often in the clinical and technical exhibits of our dental societies. It is at any rate reasonable to suppose that impressions received while seeing those clinics or reading the printed transactions, if unable to witness the clinics and listen to the discussions, has had a large place in inspiring inventions.

If then the dental society has been largely responsible for the development of dental literature and in some small degree the course of the tremendous advances in technical methods and facilities, it surely has had an important function in bringing our calling from a mere trade pursuit up to the dignity of a profession. There are those who try to look upon the practice of dentistry not as an independent self-contained profession, but if compelled to accord it the rank of other learned professions, to class it as only a medical specialty. We believe however that dentistry because of the peculiar differences which attach to its practice, and because of the great struggle by which it has come to its present acknowledged sphere of usefulness, is entitled to the rank of a useful and learned profession. It is the fact that such a high order of skill of the intellectual as well as the technical character is necessary for the practice of dentistry today that it is entitled to a place along side of other learned professions.

In 1839, when the first dental college was organized, it was assumed that a broad education was necessary to fit men for the practice of dentistry, and made proper preliminary requirements for admission, and the college made its curriculum to embrace most of the sciences of the time which had any bearing upon the human organism in health as well as disease. Since this time our college entrance requirements and their curriculums have steadily advanced until now what was some twenty-five years ago considered broad culture in science is required in every dental school in the land, and it is this that has made of us a profession.

Has the dental society been in any way responsible for the broadening of the college curriculum? We shall not attempt to show that it has brought this condition of affairs to pass entirely, but we believe an unbiased investigation will reveal the fact that such organizations have had more to do with it than any other force, not excepting the colleges themselves. If we had time to compare the events which have had most to do with the elevation of our ideals, I am confident we should find the suggestions more often made in the impromptu discussions, on the floor of dental societies of papers which are the result of careful investigation of subjects which are puzzling the general practitioners. College teachers ought to be and very frequently are advanced thinkers and workers and many of them have turned aside from their pedagogic duties to clear up by scientific research some obscure problem of practical importance, but as a rule the college teacher takes everything that has been settled to a principle and uses it not so much for the knowledge contained, but because it will assist in developing a trained mind or hand to successfully grapple with these hidden or obscure problems of daily practice. As compared with the general profession, the number of college teachers who make contributions to dental literature may be relatively large, but the practitioners as a whole make much more numerous contributions to our literature through the dental society. While the college has done well toward preparing men to practice the profession, the dental society has been after all the schoolhouse wherein has come the material advances in those things which have brought our profession to public notice. Through the societies' legislation which controls the practice in various States has been obtained, and it is well known that in almost every State and territory dental legislation which is designed to safeguard the public health from a dental standpoint is enacted on higher planes than general medical legislation. It would perhaps be tedious and tiresome to dwell further on this subject, but we can't refrain from citing the work of dental societies in securing national legislation which provides dental services for enlisted soldiers; and the efforts now ~~being~~ made to secure similar services for the men enlisted in the navy. Another philanthropic movement which has been agitated in many dental societies, and which has already been done to some extent, is the systematic inspection of the teeth of children in the public schools, with a view to securing such data as shall make it clear that something should be done in a public way to introduce proper instruction on the subject of oral hygiene into the curriculum of the public schools. Surely no nobler work or none of a more truly professional character can be cited elsewhere. It is such functions as we have enumerated that in their culmination have convinced the public that dentistry has long since ceased to be a trade and should be recognized as one of the learned as well as nobler callings of men. And there is no one factor which can be cited which has had so important a function in bringing this to pass as the dental society.

If then this organization is doing so much for the uplifting of our profession, is it not worthy of the most cordial support of every one who has chosen it as the sphere in which he is to fulfill his mission of service to his fellow men? It appeals to us as a duty, which every dentist who follows our profession because he loves it and finds in it an opportunity to serve his times advantageously, that he connect himself actively with every organizaitoon which has the capacity or gives the opportunity for enlarging his conceptions of the work he may be called upon to do. It may even be a matter of congratulation that we are privileged to meet our co-laborers under circumstances which are calculated to broaden our grasp of affairs and increase our intellectual possessions. It has always been to me a mystery how any one can absent himself from a dental society meeting simply on the plea that a social pleasure or even a business engagement prevents his attendance; or how a person can attend a meeting of dentists and come away saying that he got little or no benefit. I can not but think such a one by his own narrowness or selfishness has so dwarfed his perceptive resources that he is utterly unable to put himself in harmony with his surroundings. The person who goes to a dental society meeting and takes a rear seat and never speaks in meeting or to anyone during the intermission, and views everyone with distrust and everything that is said critically, that he may pick flaws with it, surely can not expect to realize such a degree of healthy stimulation as shall make him very well satisfied with himself or his calling. He comes to the meeting reluctantly and with no thought of gaining any great help or stimulus, and therefore ought not to be disappointed. I am happy to believe however that this is not the frame of mind which possesses those who attend dental society meetings most persistently. Such men come expecting something and many of them come ready to contribute also if the opportunity offers. It is thus that the society offers an invaluable means of stimulating progress. I have many times attended dental society meetings and gone home wholly disappointed with the results; but almost invariably this has eventually brought the stimulus needed to enable me to work out the subject under discussion, to my own satisfaction at least.

One gets at a subject from so many view points in these free discussions that he can not become narrow if he would. In this open meeting there can be no such thing as for one to force an erroneous view by the subtility of his eloquence or logic; because there is likely always to be some one present who has gone over the same ground, possibly by a different route, and reached a different conclusion. It is this rebuttal testimony or experience which keeps us from bigotry and error.

Viewed from this standpoint is not a dental society a much needed and when wisely conducted an invaluable means of culture for the individual as well as a means of disseminating technical progress? We might very well make a positive assertion of its value for such a purpose, and also strengthen the assertion by the additional statement that there has not yet appeared another organization which can take its place. It is unique in its function; and because of the versatility of its application the society or convention method of accomplishing its purpose is being almost universally adopted as the best way of securing rapid and economical exchange of ideas. While much that is said or done in the impromptu proceeding of societies is either flippant or irrelevant, and quite a little of erroneous doctrine may creep in insiduously or accidentally, yet the general output can be depended upon as a fair representation of the activities of the profession. The new things are of course always brought out in conventions, and many repetitions of old, and sometimes forgotten ideas which are good and worthy of restatement, are brought out for wholesome reconsideration.

May we not from even this hurried review of its work and power, conclude that the function and value of the dental society is to supply the best means of personal and professional advancement, and that its value may be gaged by the earnestness and ability of its membership?

